# 3,5,6,7,8,3′,4′-Heptamethoxyflavone Ameliorates Depressive-Like Behavior and Hippocampal Neurochemical Changes in Chronic Unpredictable Mild Stressed Mice by Regulating the Brain-Derived Neurotrophic Factor: Requirement for ERK Activation

**DOI:** 10.3390/ijms18102133

**Published:** 2017-10-12

**Authors:** Atsushi Sawamoto, Satoshi Okuyama, Yoshiaki Amakura, Morio Yoshimura, Takashi Yamada, Hidehiko Yokogoshi, Mitsunari Nakajima, Yoshiko Furukawa

**Affiliations:** 1 Department of Pharmaceutical Pharmacology, Graduate school of Clinical Pharmacy, Matsuyama University, 4-2 Bunkyo-cho, Matsuyama, Ehime 790-8578, Japan; 46140018@g.matsuyama-u.ac.jp (A.S.); sokuyama@g.matsuyama-u.ac.jp (S.O.); mnakajim@g.matsuyama-u.ac.jp (M.N.); 2 Department of Pharmacognosy, Graduate school of Clinical Pharmacy, Matsuyama University, 4-2 Bunkyo-cho, Matsuyama, Ehime 790-8578, Japan; amakura@g.matsuyama-u.ac.jp (Y.A.); myoshimu@g.matsuyama-u.ac.jp (M.Y.); 3 Department of Food and Nutritional Sciences, College of Bioscience and Biotechnology, Chubu University, 1200 Matsumoto-cho, Kasugai, Aichi 487-8501, Japan; yamadat@nagoya-ku.ac.jp (T.Y.); yokogoshi@isc.chubu.ac.jp (H.Y.)

**Keywords:** 3,5,6,7,8,3′,4′-heptamethoxyflavone, HMF, depression, brain-derived neurotrophic factor, neurogenesis, neuroplasticity, astrocyte

## Abstract

We previously reported that the subcutaneous administration of 3,5,6,7,8,3′,4′-heptamethoxyflavone (HMF), a citrus polymethoxyflavone, attenuated depressive-like behavior and increased the expression of brain-derived neurotrophic factor (BDNF) in the hippocampus of a corticosterone-induced depression-like mouse model. We herein demonstrated that (1) HMF was detectable in the brain 10 and 30 min after its oral administration, (2) orally administered HMF improved chronic unpredictable mild stress (CUMS)-induced pathological conditions, including body weight loss and depressive-like behavior, and CUMS-induced neurochemical changes, such as reduction in BDNF expression, decrease in neurogenesis, and decreased level of phosphorylated calcium-calmodulin-dependent protein kinase II in the hippocampus, and (3) these effects of HMF were inhibited by the pre-administration of U0126, a mitogen-activated protein (MAP) kinase inhibitor. These results suggest that orally administered HMF is beneficial for the upregulation of BDNF in the hippocampus via the extracellular signal-regulated kinase1/2 (ERK1/2)/MAP system, which may account for its antidepression effects.

## 1. Introduction

Brain-derived neurotrophic factor (BDNF) is the most widely distributed neurotrophic factor in the central nervous system (CNS), and it plays key roles in plasticity, survival, and neurogenesis in adult brains as well as in growth and differentiation in developing brains [1]. The “BDNF hypothesis of depression”, which states that BDNF is involved in the pathogenesis of depression [2,3], is based on findings showing that (1) long-term stress activates the hypothalamic-pituitary-adrenal (HPA) axis, resulting in the excess secretion of glucocorticoids [4,5], which is linked to the suppressed production of BDNF [6,7,8,9,10]; (2) BDNF levels in serum/plasma are reduced in depressed patients, and this is attenuated by antidepressants [11]; (3) BDNF levels are reduced in the postmortem brains of suicides [12]; and (4) reduced brain levels of BDNF contribute to atrophy and cell loss in the hippocampus and prefrontal cortex, as observed in depressed subjects [13].

Based on these findings, a rodent model of depression has been developed by the chronic administration of corticosterone (CORT) [14,15], a prominent glucocorticoid. Using these CORT-treated mice, we previously revealed that the repeated subcutaneous (*s.c.*) administration of 3,5,6,7,8,3′,4′-heptamethoxyflavone (HMF), a polymethoxyflavone contained in citrus peel, ameliorated (1) CORT-induced reductions in BDNF levels in the hippocampus; (2) the CORT-induced inactivation of astrocytes, resulting in a decrease in the expression of BDNF; (3) CORT-induced reductions in neurogenesis in the hippocampus; (4) CORT-induced reductions in the expression levels of phosphorylated calcium-calmodulin-dependent protein kinase (p-CaMK) II in the hippocampus; (5) CORT-induced reductions in body weight gain, and (6) CORT-induced depressive-like behavior [16]. We also showed that HMF administered intraperitoneally (*i.p.*) to mice was immediately detected in the brain [17]. These findings suggest that HMF functions as an antidepressant drug via the induction of BDNF expression [16].

In order to develop antidepressant drugs from HMF based on the “BDNF hypothesis of depression”, we need to investigate (1) whether orally (*p.o.*) administered HMF enters the brain; (2) whether HMF induces BDNF expression in a more physiological mouse model of depression than CORT-treated mice; and (3) the mechanisms of action of HMF. Regarding (1), we changed the administration route of HMF from *s.c.* to *p.o.* in the present study, and performed a quantitative analysis of HMF in the brain using liquid chromatography/mass spectrometry (LC/MS). Concerning (2), we used the “chronic unpredictable mild stress (CUMS)-induced depression-like mouse model” in the present study. CUMS-treated mice or rats have frequently been used as a prototypical model of depression [18,19,20]. In this model, rodents are continuously and unpredictably exposed to a series of mild stressors such as the deterioration of house conditions, starvation, and physical restraint. The neurobiological abnormalities of CUMS-treated rodents are reported to be similar to those in depressed patients, such as decreases in hippocampal BDNF or the suppression of neurogenesis [21,22]. Regarding (3), we focused on the extracellular signal-regulated kinase1/2 (ERK1/2)-mitogen-activated protein kinase (MAP) pathway. ERK1/2, which is downstream of several receptors and a regulator of the transcription of many targets, has been implicated in the depression-like symptoms elicited by stress-related insults, and antidepressants have been shown to restore stress-related reductions in hippocampal phosphorylated ERK1/2 (p-ERK1/2; the activated form of ERK1/2) [14]. We previously indicated that HMF has the ability to activate (phosphorylate) ERK1/2 in cultured neurons and that the pretreatment of neurons with a MAP kinase (MAPK)/ERK kinase (MEK) inhibitor (either U0126 or PD98059) significantly abolished the HMF-induced increase in p-ERK [23]. We also showed that HMF has the ability to attenuate CORT-induced decrease in p-ERK1/2 in the hippocampus [16], and that HMF has the ability to attenuate MK-801-induced decrease in p-ERK1/2 in the hippocampus, which was suppressed with the pretreatment of U0126 [17]. Therefore, we herein used U0126 to investigate the mechanisms of action of HMF.

## 2. Results

### 2.1. Assessment of Brain Levels of 3,5,6,7,8,3′,4′-Heptamethoxyflavone (HMF) after Its Oral Administration

**Figure 1:**
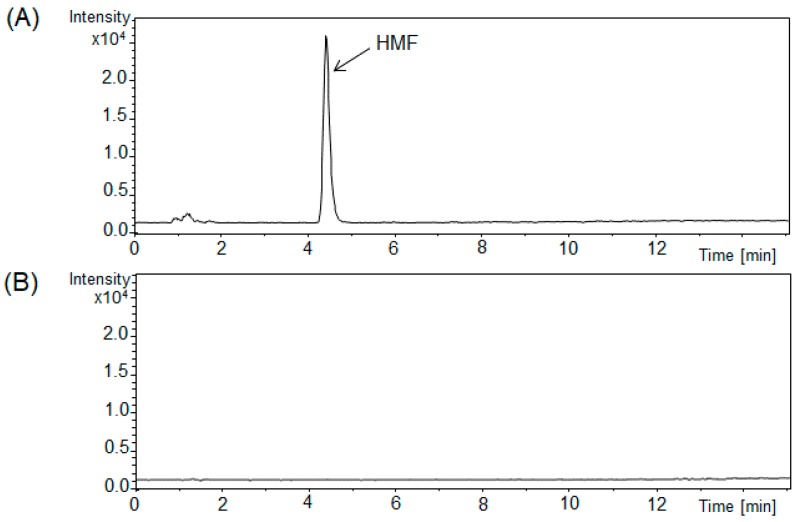
Liquid chromatography/mass spectrometry (LC/MS) chromatograms (*m*/*z* 433 [M + H]^+^) of brain tissues from mice treated orally with 3,5,6,7,8,3′,4′-heptamethoxyflavone (HMF) (**A**) and vehicle-treated brains (**B**). The solid arrow is HMF.

In order to investigate whether HMF administered *p.o.* passes through the blood–brain barrier (BBB), we developed a more sensitive LC/MS method to measure a very small quantity of polymethoxyflavone in brain tissues than the previous analytical high performance liquid chromatographic (HPLC) method [17]. Mice were administered HMF (100 mg/kg) *p.o.* once, and brain samples were analyzed using LC/MS conditions 10, 30, 60, and 90 min after its administration. Figure 1A shows typical chromatograms of HMF-treated brain samples, revealing a sharp peak in HMF. The calibration curve for the standard of HMF was linear over the range of 0.01–1 μg/mL. The correlation coefficient (*r*) was >0.999, as obtained by the analysis, suggesting good linearity between the peak area ratio and HMF concentrations. Figure 1B shows typical chromatograms of non-treated brain samples, revealing the absence of interfering peaks.

**Figure 2:**
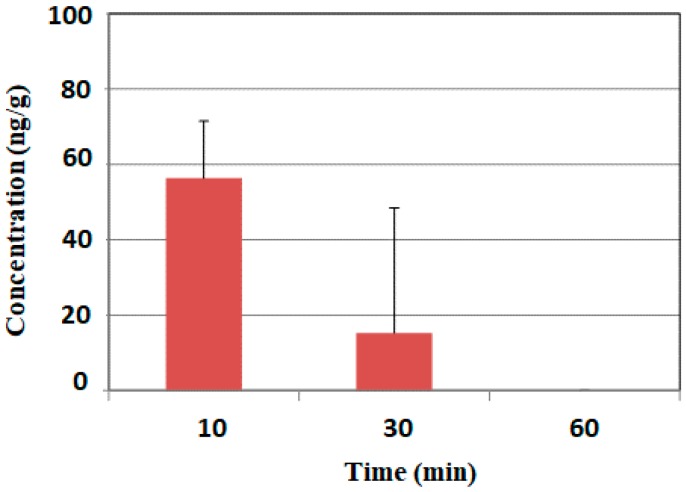
Time course of brain HMF concentration profiles following its *p.o.* administration. Values are means ± SEM (*n* = 3).

Figure 2 shows the mean concentration-time profile of HMF in the brain. The content of HMF in the brain reached its highest level (56.4 ± 15.1 ng/g) 10 min after its administration, markedly declined to 15.3 ± 33.2 ng/g by 30 min, and reached trace levels by 60 and 90 min. These results indicated that HMF was quickly absorbed.

### 2.2. Effects of Chronic Unpredictable Mild Stress (CUMS) and HMF Treatments on Pathological Conditions

Mice were divided into five experimental groups: an unstressed normal situation group as control group (CON group, *n* = 12), CUMS-loaded control group (CUMS group, *n* = 16), CUMS-loaded plus low-dose HMF-treated (50 mg/kg) group (CUMS + HMF-L group, *n* = 16), CUMS-loaded plus high-dose HMF-treated (100 mg/kg) group (CUMS + HMF-H group, *n* = 16), and CUMS-loaded plus high-dose HMF-plus U0126-treated group (CUMS + HMF-H + U0126 group, *n* = 16).

**Figure 3:**
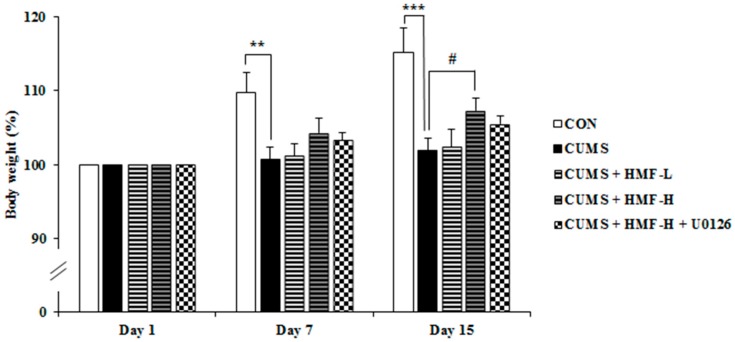
Effects of chronic unpredictable mild stress and HMF treatments on body weight changes on Days 1, 7, and 15. Values are means ± SEM (*n* = 12–16). Symbols show significant differences between the following groups: CON vs. CUMS (** *p* < 0.01, *** *p* < 0.001, the Student’s *t*-test) and CUMS vs. CUMS + HMF-H (^#^ *p* < 0.05, one-way ANOVA followed by the post-hoc Williams multiple comparison test).

The measurement of body weight is usually used as a nonspecific indicator of poor health in rodents. We thus measured body weight three times during the experiment period (on Days 1, 7, and 15). At the beginning of the experiment (Day 1), the average body weights of mice were 21.5 ± 0.6 g in the CON group, 22.6 ± 0.5 g in the CUMS group, 22.0 ± 0.4 g in the CUMS + HMF-L group, 21.4 ± 0.4 g in the CUMS + HMF-H group, and 21.2 ± 0.2 g in the CUMS + HMF-H + U0126 group, which were defined as 100%, respectively. As shown in Figure 3, body weight in the CON group increased during the experimental period of 2 weeks; 110 ± 1.7% on Day 7 and 115 ± 3.3% on Day 15. Consistent with previous findings [24], no marked increases were observed in body weight in the CUMS group. The statistical analysis revealed that body weights in the CUMS group on Day 7 (101 ± 1.7%) and Day 15 (110 ± 2.8%) were significantly (** *p* < 0.01 and *** *p* < 0.001, respectively) lower than those in the CON group. Among the four CUMS groups (CUMS, CUMS + HMF-L, CUMS + HM-H, and CUMS + HMF-H + U0126 group), no significant differences were observed on Day 7. On Day 15, body weight in the CUMS + HMF-H group (107 ± 1.8%) was significantly (^#^ *p* < 0.05) higher than that in the CUMS group, suggesting that HMF has the ability to ameliorate CUMS-induced reductions in body weight gain.

**Figure 4:**
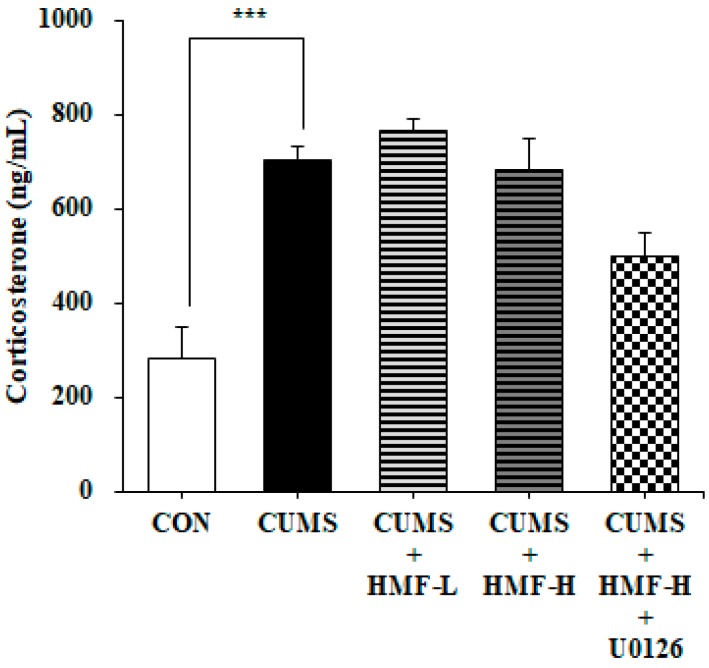
Effects of HMF on serum corticosterone levels on Day 16. Values are means ± SEM (*n* = 6–8). *** *p* < 0.001, CON vs. CUMS (the Student’s *t*-test).

At the end of the experiment (on Day 16), serum CORT levels were measured. Figure 4 shows that serum CORT concentrations in the CUMS group (705 ± 64.3 ng/mL) were significantly (*** *p* < 0.001) higher than those in the CON group (284 ± 27.9 ng/mL), as previously reported [25]. Figure 4 also shows that there were no significant differences between the CUMS and CUMS + HMF groups, suggesting that HMF does not ameliorate CUMS-induced increases in serum CORT concentrations.

### 2.3. Effects of HMF on Depressive-Like Behavior in CUMS-Treated Mice

The forced swim test (FST) is a commonly used technique to screen antidepressants and assess depressive-like behavior [26,27]. We previously demonstrated that the immobility time in FST in the CORT-injected group was approximately twice that in the CON group [16]. We confirmed in our preliminary studies that the immobility time of the CUMS-treated group was approximately twice that of the CON group at Day 11 in the present experimental system. Based on this result, we did not measure the immobility time of the CON group in this experiment because FST at Day 15–16 may affect serum CORT concentrations.

**Figure 5:**
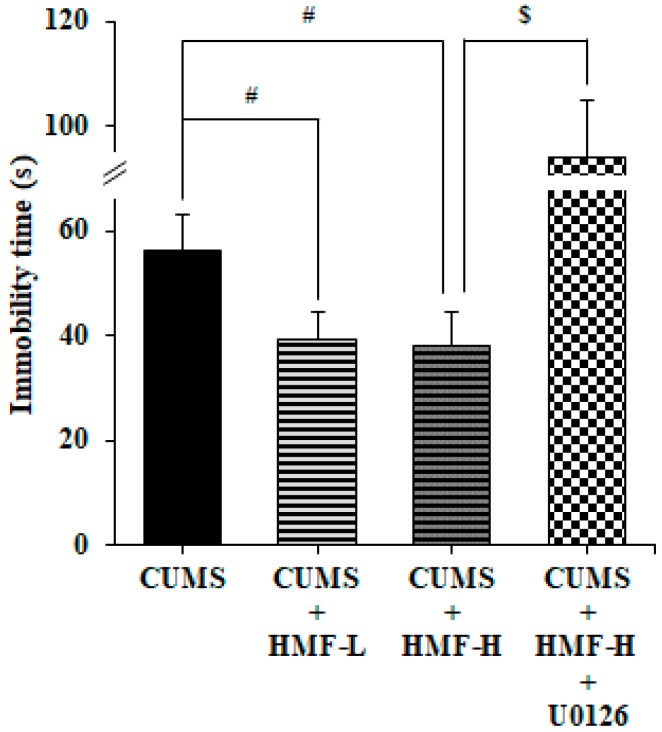
Effects of HMF on CUMS-induced depression-like behavior in the forced swim test on Day 16. Values are means ± SEM (*n* = 15–16). Symbols show significant differences between the following groups: CUMS vs. CUMS + HMF-L, CUMS vs. CUMS + HMF-H (^#^ *p* < 0.05, one-way ANOVA followed by the post-hoc Williams multiple comparison test) and CUMS + HMF-H vs. CUMS + HMF-H + U0126 (^$^ *p* < 0.05, the Student’s *t*-test).

Figure 5 shows that immobility times in FST in the CUMS + HMF-L group (39.3 ± 5.3 s) and CUMS + HMF-H group (38.2 ± 6.4 s) were significantly (^#^ *p* < 0.05) shorter than that in the CUMS group (56.2 ± 7.0 s), indicating that HMF has the ability to ameliorate depressive-like behavior. Figure 5 also shows that the immobility time in FST in the CUMS + HMF-H + U0126 group (94.3 ± 5.6 s) was significantly (^$^ *p* < 0.05) higher than that in the CUMS + HMF-H group, suggesting that the ameliorative effects of HMF on depressive-like behavior were inhibited by U0126.

### 2.4. Effects of CUMS and HMF Treatments on Hippocampal BDNF Protein Expression

**Figure 6:**
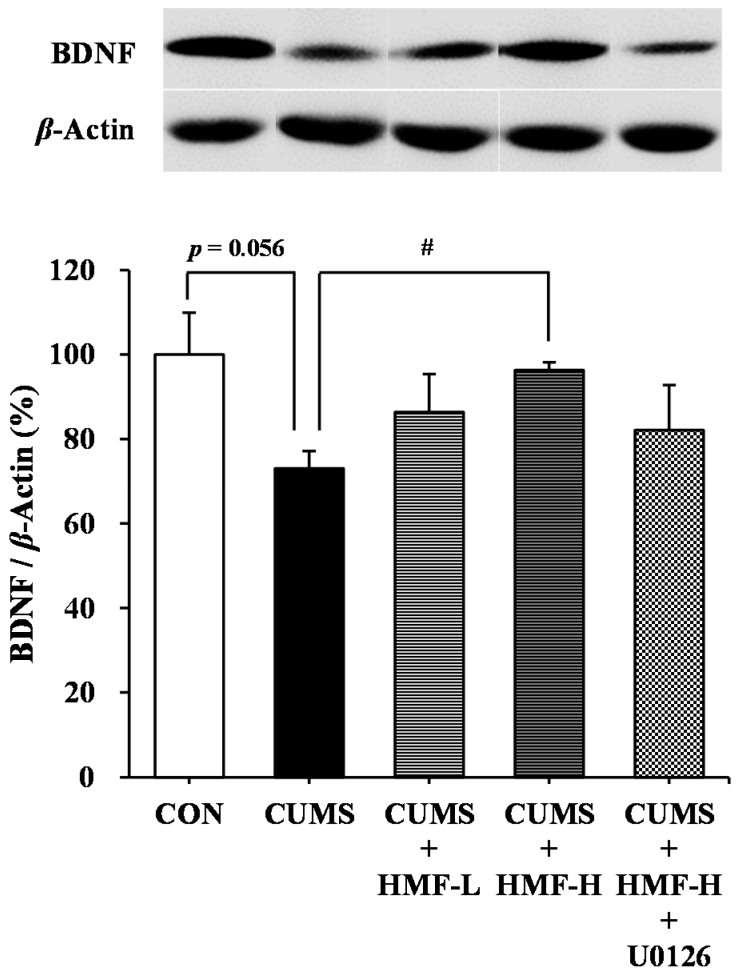
Western blot analysis of brain-derived neurotrophic factor (BDNF) in the CUMS-treated mouse hippocampus on Day 16. Values are means ± SEM (*n* = 5). CON vs. CUMS (*p* = 0.056 the Student’s *t*-test), CUMS vs. CUMS + HMF-H (^#^ *p* < 0.05, one-way ANOVA followed by the post-hoc Williams multiple comparison test).

We investigated the effects of HMF on BDNF expression in the mouse hippocampus on Day 16 using a western blot analysis. As shown in Figure 6, BDNF protein levels in the CUMS group (73.0 ± 4.2%) was slightly (*p* = 0.056) lower than those in the CON group (100 ± 9.9%). This reduction in BDNF was significantly (^#^ *p* < 0.05) suppressed in the CUMS + HMF-H group (96.4 ± 1.8%), suggesting that HMF has the ability to ameliorate CUMS-induced decreases in BDNF levels in the hippocampus. The reviving effects of HMF on the downregulated expression of BDNF were not inhibited by U0126 contrary to our expectations.

**Figure 7:**
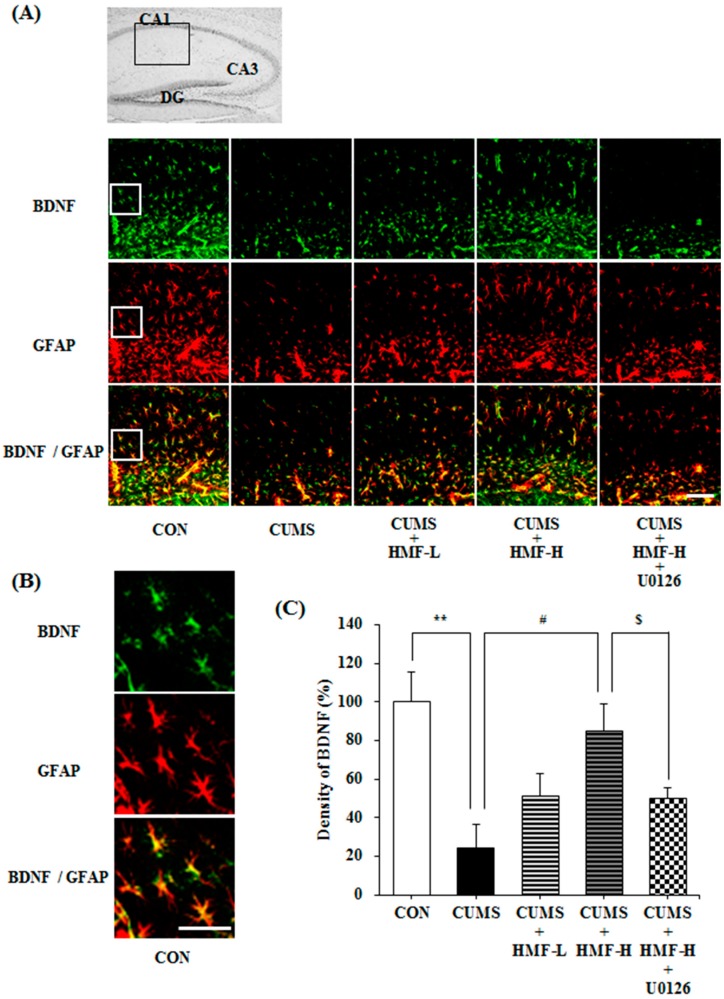
Effects of HMF on the expression of BDNF and glial fibrillary acidic protein (GFAP) in the CUMS-treated mouse hippocampus on Day 16. (**A**) Immunoreactivities of BDNF (green) and GFAP (red) with lower magnification. Merged images were shown in yellow. The white scale bar shows 100 μm. The portion for the quantitative analysis was shown with a black square. (**B**) Immunoreactivities of BDNF (green) and GFAP (red) with higher magnification. Merged images were shown in yellow. The white scale bar shows 100 μm. The portion for the enlargement was shown with a white square in (**A**). (**C**) A quantitative analysis of BDNF densities of the total areas of (**A**). Values are means ± SEM (*n* = 6–8). Symbols show significant differences between the following groups: CON vs. CUMS (** *p* < 0.01, the Student’s *t*-test), CUMS vs. CUMS + HMF-H (^#^ *p* < 0.05, one-way ANOVA followed by the post-hoc Williams multiple comparison test) and CUMS + HMF-H vs. CUMS + HMF-H + U0126 (^$^ *p* < 0.05, the Student’s *t*-test). CA, cornu ammonis; DG, dentate gyrus.

As the specificity of the anti-BDNF antibody was confirmed by western blot analysis, we then investigated the effects of HMF on BDNF expression in mouse hippocampus regions (the stratum lacunosum-moleculare of Ammon’s horn and molecular layer of the dentate gyrus; DG) using an immunohistochemical method, a valuable tool for detecting specific antigens in tissues. Figure 7A,B show representative photographs, demonstrating that (1) the BDNF signal (green signal) in the CUMS group was markedly weaker than that in the CON group; (2) this signal in the CUMS + HMF-L group and CUMS + HMF-H group was stronger than that in the CUMS group; and (3) this signal in the CUMS + HMF-H + U0126 group was weaker than that in the CUMS + HMF-H group. Figure 7A,B show that the BDNF signal and that of glial fibrillary acidic protein (GFAP; a marker of activated astrocytes; red signal) in each group overlapped with each other (yellow signal). A quantitative analysis of BDNF-positive cell densities (Figure 7C) showed that the density of the BDNF signal in the CUMS group (24.4 ± 11.9%) was significantly (** *p* < 0.01) weaker than that in the CON group (100.0 ± 15.7%). This decrease in the BDNF signal was suppressed in the CUMS + HMF-L group (51.6 ± 11.0%) and CUMS + HMF-H group (85.3 ± 13.5%, ^#^ *p* < 0.05), suggesting that HMF has the ability to ameliorate CUMS-induced decreases in BDNF expression in the hippocampus. Figure 7C also shows that BDNF signal density in the CUMS + HMF-H + U0126 group was 50.3 ± 5.4% that in the CON group, indicating that this effect of HMF was significantly (^$^ *p* < 0.05) inhibited by U0126. The reason why there was difference between the data of western blot analysis and that of immunohistochemical analysis might be due to the difference of the sensitivities of the two methods.

### 2.5. Effects of CUMS and HMF Treatments on Hippocampal Neurogenesis

**Figure 8:**
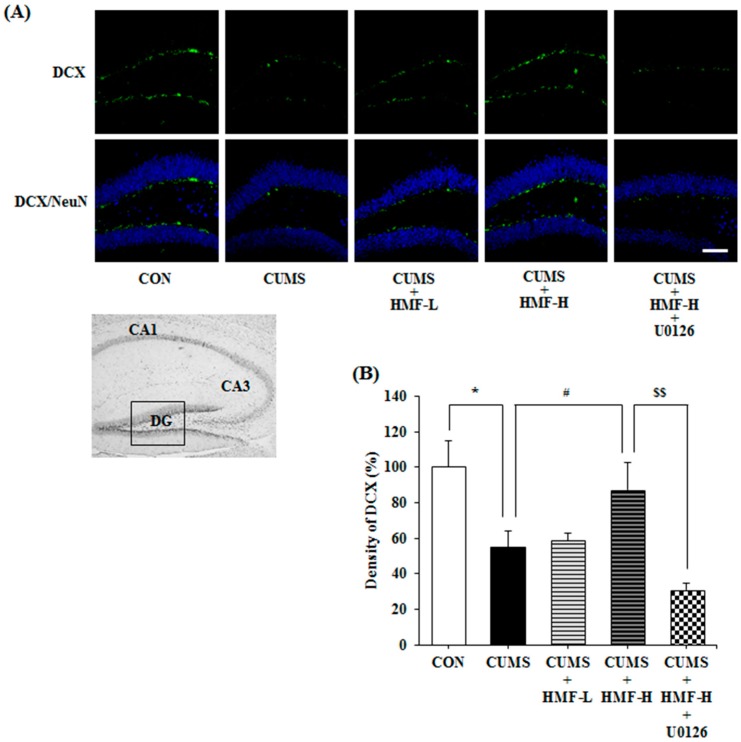
Effects of HMF on neurogenesis in the CUMS-treated mouse hippocampus on Day 16. (**A**) Immunoreactivities of doublecortin (DCX) (green) and neuronal nuclei (NeuN) (blue). The white scale bar shows 100 μm. The portion for the quantitative analysis was shown with a black square. (**B**) A quantitative analysis of DCX densities. Values are means ± SEM (*n* = 6–8). Symbols show significant differences between the following groups: CON vs. CUMS (* *p* < 0.05, the Student’s *t*-test), CUMS vs. CUMS + HMF-H (^#^ *p* < 0.05, one-way ANOVA followed by the post-hoc Williams multiple comparison test) and CUMS + HMF-H vs. CUMS + HMF-H + U0126 (^$$^ *p* < 0.01, the Student’s *t*-test).

We then investigated the effects of the *p.o.* administration of HMF on neurogenesis in CUMS-treated mice on Day 16. Figure 8A shows representative photographs of the hippocampal DG region stained with specific antibodies for doublecortin (DCX; a marker for immature neuronal cells; green signal) and neuronal nuclei (NeuN; a marker for microtubule-associated protein expressed by neuronal precursor cells; blue signal). Figure 8B is a quantitative analysis of DCX densities, showing that the density of the DCX signal in the CUMS group (55.1 ± 8.8%) was significantly (* *p* < 0.05) weaker than that in the CON group (100 ± 15.1%), as previously reported [28]. This decrease in the DCX signal in the DG region was significantly (^#^ *p* < 0.05) suppressed in the CUMS + HMF-H group (87.4 ± 15.2%), suggesting that HMF has the ability to ameliorate CUMS-induced decreases in neurogenesis in the hippocampus. Figure 8A,B also show that the effects of HMF were significantly (^$$^ *p* < 0.01) suppressed by U0126 (density in the CUMS + HMF-H + U0126 group was 31.0 ± 3.7%).

### 2.6. Effects of CUMS and HMF Treatments on the Levels of Phosphorylated Calcium-Calmodulin-Dependent Protein Kinase (p-CaMK) II

**Figure 9:**
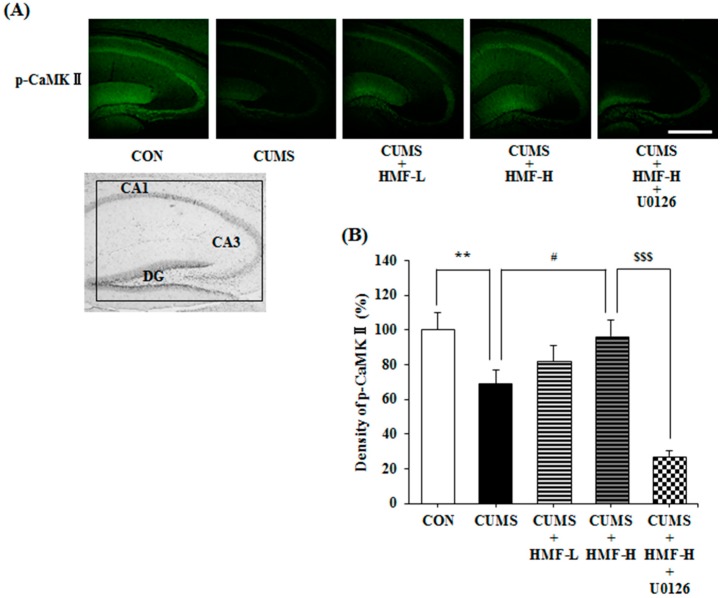
Influence of HMF on the levels of p-CaMK II in the CUMS-treated mouse hippocampus on Day 16. (**A**) Immunoreactivity of phosphorylated calcium-calmodulin-dependent protein kinase (p-CaMK) II (green). The white scale bar shows 500 μm. The portion for the quantitative analysis was shown with a black square. (**B**) A quantitative analysis of p-CaMK II densities. Values are means ± SEM (*n* = 6–8). Symbols show significant differences between the following groups: CON vs. CUMS (** *p* < 0.01, the Student’s *t*-test), CUMS vs. CUMS + HMF-H (^#^ *p* < 0.05, one-way ANOVA followed by the post-hoc Williams multiple comparison test) and CUMS + HMF-H vs. CUMS + HMF-H + U0126 (^$$$^ *p* < 0.001, the Student’s *t*-test).

CaMK II, one of the serine/threonine protein kinases existing globally in the CNS, is known to play a crucial role in the induction and maintenance of long-term potentiation (LTP) in the hippocampus, and its auto-phosphorylation relates to neuroplasticity [29]. Therefore, we examined the influence of CUMS treatments and the *p.o.* administration of HMF on the levels of p-CaMK II in the mouse hippocampus with an immunohistochemical method using a specific antibody against p-CaMK II. Figure 9A shows representative photographs of the hippocampal region stained with anti-p-CaMK II antibody (green signal) on Day 16. Figure 9B is a quantitative analysis of p-CaMK II densities. The density of the p-CaMK II signal in the CUMS group (69.2 ± 7.8%) was significantly (** *p* < 0.01) weaker than that in the CON group (100 ± 10.1%). This decrease was significantly (^#^ *p* < 0.05) suppressed in the CUMS + HMF-H group (96.7 ± 9.4%), suggesting that HMF has the ability to ameliorate CUMS-induced decreases in the levels of p-CaMK II in the hippocampus. Figure 9A,B also show that the effects of HMF were significantly (^$$$^ *p* < 0.001) suppressed by U0126 (density in the CUMS + HMF-H + U0126 group was 27.2 ± 3.5%).

## 3. Discussion

We previously reported that HMF-injected *i.p.* was immediately detected in the mouse brain [17]. In the present study, we successfully showed that HMF-administered *p.o.* was also immediately detected in the mouse brain (Figure 2), suggesting that HMF-administered *p.o.* passes quickly through the BBB, similar to that administered *i.p.*

We also previously demonstrated that HMF administered *s.c.* exerted antidepressant-like effects in a CORT-induced depression-like mouse model, and suggested that the effects of HMF were due to its ability to induce BDNF [16]. In the present study, we successfully showed that HMF administered *p.o.* also ameliorated CUMS-induced depression-like pathologies (body weight loss; Figure 3, and prolonged immobility in FST; Figure 5) and hippocampal reductions in BDNF expression (Figure 6 and Figure 7). These results support our suggestion [16] that HMF functions as an antidepressant drug via the induction of BDNF expression. Previous studies indicated that hippocampal astrocytes are crucial regulators of CNS development, function, and health [30], and that they also promote the synthesis of BDNF in response to antidepressants [31]. Consistent with these findings, Figure 7 shows that the BDNF-positive signal nearly merged with the GFAP-positive signal, suggesting that the site of BDNF expression is astrocytes.

BDNF expression in the hippocampus has been assumed to increase with the chronic administration of antidepressant drugs [32], and is followed by the proliferation and differentiation of neuronal progenitor cells [28,33,34] and restoration of neuronal plasticity [35,36]. In accordance with these findings, Figure 8 shows that CUMS induced the downregulation of neurogenesis in the hippocampal DG, and also that HMF treatments suppressed this phenomenon. Figure 9 shows that CUMS reduced p-CaMK II in the hippocampus, and that HMF treatments suppressed this phenomenon. These results in combination with our previous findings [16] strongly indicate that HMF is a novel antidepressant based on the “BDNF theory of depression”.

Regarding the mechanisms of action of HMF, we demonstrated that the effects of HMF on CUMS-induced behavioral and neurochemical changes were abolished by U0126 treatments (Figure 5, Figure 7, Figure 8 and Figure 9), suggesting that the antidepressant-like effects of HMF are mediated by the activation of MEK. According to our findings, BDNF binds to tropomyosin receptor kinase B (TrkB), the high affinity receptor for BDNF, and BDNF-TrkB activates (phosphorylates) ERK1/2 via the Ras-Raf-MEK-ERK cascade [37], which mediates survival, growth, differentiation, plasticity, and anti-depression [13,38]. In contrast, we previously reported that HMF has the ability to promptly activate (phosphorylate) not only ERK1/2, but also cyclic AMP (cAMP) response element-binding protein (CREB) in cultured neurons [23]. CREB is a transcription factor that plays a critical role in various neuronal functions, such as the regulation of learning and memory, and is activated by ERK [39]. BDNF is one of the downstream targets of phosphorylated CREB [40]. Therefore, there is a dual and directional regulatory mechanism between BDNF/TrkB and ERK/CREB. Another citrus polymethoxyflavone, 5,6,7,8,3′,4′-hexamethoxyflavone (nobiletin) has been suggested to stimulate cAMP/cAMP-dependent protein kinase (PKA)/ERK/CREB signaling associated with learning and memory in cultured neurons [41,42]. Macranthol, a natural lignin, has been demonstrated to exert antidepressant-like effects in mice, at least in part, by increasing hippocampal BDNF expression via the BDNF/TrkB-PI3K/Akt signaling pathway [43]. These findings indicate that HMF activates ERK/CREB signaling, followed by the induction of BDNF, as well as BDNF/TrkB signaling via ERK signaling. We are currently examining the mechanisms of action of HMF in vitro.

Chronic stress is known to induce increases in serum/plasma glucocorticoid levels (cortisol in humans and CORT in rodents), which are associated with metabolic disturbances [44]. Acute stress by FST was also reported to increase the serum CORT concentrations [45]. Figure 4 shows that HMF did not ameliorate CUMS-induced increases in serum CORT concentrations. In accordance with our previous result [16], the antidepressant-like actions of HMF might be not directly related to the modulation of HPA axis activity.

## 4. Materials and Methods

### 4.1. Animals

Male C57BL/6 strain mice (seven weeks old) were purchased from Japan SLC, Inc. (Hamamatsu, Shizuoka, Japan). Stock diets and tap water were freely available during the experimental period. Mice were kept at 23 ± 1 °C on a 12-h light/dark cycle (lights on 8:00–20:00). All animal experiments were performed in accordance with the Guidelines for Animal Experimentation and Approved Protocol (#14005, 10 October 2014) by the Animal Care and Use Committee of Matsuyama University.

### 4.2. Brain Sample Preparation for the LC/MS Analysis

HMF was kindly gifted from Ushio ChemiX Corp. (Omaezaki, Japan). HMF was dissolved in 5% DMSO in saline, and *p.o.* administered (100 mg/kg) to mice (*n* = 3 at each point). At 10, 30, 60 and 90 min after its administration, mice were deeply anesthetized with diethyl ether, perfused with ice-cold phosphate-buffered saline (PBS) through the left ventricle, and the brains were excised. In order to prepare samples for the LC/MS analysis, brain samples were sonicated as previously described [17].

### 4.3. Quantitative Analysis of HMF via LC/MS

The LC/MS analysis was performed using the Agilent 1200 Series HPLC system (Agilent Technologies, Santa Clara, CA, USA) equipped with a micrOTOF-Q Mass Spectrometer (Bruker Daltonics, Billerica, MA, USA). LC conditions were as follows: column, L-column 2 ODS (150 mm × 2.1 i.d., 5 μm; Chemicals Evaluation and Research Institute, Tokyo, Japan) mobile phase, 0.1% formic acid in water (A) 0.1% formic acid in acetonitrile (B) in a gradient mode of 50% B (0 min) → 100% B (15 min); injection volume, 2 μL; flow rate, 0.2 mL/min. MS conditions were as follows: interface, ESI positive; capillary, 4500 eV; nebulizer, 400 hPa; dry gas, 4.0 L/min; dry temperature, 180 °C; collision energy, 12.0 eV; transfer time, 90 μs; MS range, *m*/*z* 433 (Selective ion monitoring (SIM)).

### 4.4. CUMS Procedure

The CUMS procedure was performed with a minor modification to the methods reported previously [18,19]. Stress paradigms in this study consisted of six stressors including a forced swim stress (15 min), wet sawdust stress (24 h), food deprivation stress (24 h), restraint stress (2 h), cage tilt stress (30°, 24 h), foot-shock stress (intensity 0.5 mA, duration 5 s, intershock interval 30 s, 20 shocks). One of these stressors was given in a random order for 14 days (Table 1). All stressors were started at 10:00–11:00 a.m. after the drug treatment. Mice in the CON group were housed in a separate room and had no contact with the CUMS-loaded groups.

### 4.5. Drug Treatment of CUMS-Treated Mice

**Figure 10:**
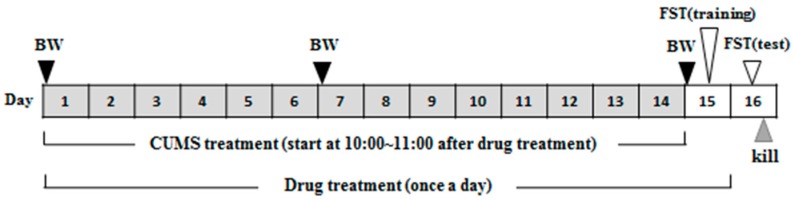
Schematic drawing of experimental schedule. BW means measurement of body weight.

Experimental protocol for drug treatment is shown in Figure 10. Mice in the CUMS + HMF-L group were orally administered HMF (50 mg/kg) once a day for 15 days. Mice in the two CUMS-loaded plus high-dose HMF-treated groups (CUMS + HMF-H group and CUMS + HMF-H + U0126 group) were orally administered HMF (100 mg/kg) once a day for 15 days. Mice in the CON group and mice in the CUMS group were orally administered vehicle (5% DMSO in saline; 20 mL/kg) once a day for 15 days.

U0126 was purchased from Wako Pure Chemical Industries, Ltd. (Osaka, Japan) and dissolved in 1% DMSO in saline. In the CUMS + HMF-H + U0126 group, U0126 (0.4 mg/kg) was *i.p.* administered to mice 30 min before the daily oral administration of HMF during the experimental period.

### 4.6. FST

The FST was performed according to the standard protocol [26] with minor modifications. In brief, mice were individually placed at the center of a plastic cylinder (height 27 cm × diameter 18 cm) filled with water (up to 18 cm high) at 24 ± 1 °C, and were allowed to swim freely for 10 min on Day 15 (training session) and 5 min on Day 16 (test session) as shown in Figure 10. The duration of immobility during the first 3 min of the test was recorded by the ANY-maze Video Tracking System (Stoelting, Wood Dale, IL, USA) connected to a USB digital camera.

### 4.7. Measurement of Serum CORT Concentrations

Blood was collected from decapitated mice into 1.5-mL tubes immediately after the FST. Blood was then left at 4 °C for 24 h, and centrifuged at 1200× *g* at 4 °C for 15 min to collect serum. Serum CORT concentrations were measured using a Corticosterone ELISA Kit (Assaypro LLC, St. Charles, MO, USA).

### 4.8. Western Blot Analysis

In order to collect brain tissues for the immunohistochemical and western blot analyses, mice were transcardially perfused with ice-cold PBS immediately after the FST. For each brain of 5 mice, tissue extracts were prepared as described previously [46], and equal amounts of protein (50 μg) were applied for the immunoblot analysis. The specific antibodies used in this study were rabbit antibodies against BDNF (dilution 1:1000, Abcam, Cambridge, UK) and rabbit antibodies against β-actin (1:1000, Sigma-Aldrich, St. Louis, MO, USA). The secondary antibody was horseradish peroxidase-linked anti-rabbit IgG (Cell Signaling, Denver, MA, USA). Immunoreactive bands were visualized by ECL-prime (GE Healthcare, Chalfont St. Giles, UK), and band intensities were measured using a LAS-3000 imaging system (Fujifilm, Tokyo, Japan). The quantitative analysis of band intensities was conducted using ImageJ software (NIH, Bethesda, Rockville, MD, USA), and data were normalized with β-actin.

### 4.9. Immunohistochemistry

For each brain of four mice, at least six sections were selected for analysis. The protocol for the analysis was performed as previously described [16]. Sagittal sections at a thickness of 30 μm were stained with the following primary antibodies: rabbit anti-BDNF (dilution 1:150; Abcam), mouse anti-GFAP (1:200; Sigma-Aldrich), goat anti-DCX (1:50; Santa Cruz Biotechnology, Santa Cruz, CA, USA), mouse anti-NeuN (1:300; Millipore, Billerica, MA, USA), and rabbit anti-phospho CaMK II (p-Thr286, 1:500; Sigma-Aldrich). As secondary antibodies, Alexa Fluor 568 Goat anti-Mouse IgG (1:300; Invitrogen, Carlsbad, CA, USA), Alexa Fluor 488 Goat anti-Rabbit IgG (1:300; Invitrogen), and Alexa Fluor 488 Donkey anti-Goat IgG (1:300; Invitrogen) were used. Stained sections were enclosed by mounting medium with DAPI (Vectashield; Vector Laboratories, Burlingame, CA, USA), and images were captured with a confocal fluorescence microscopy system (LSM510; Zeiss, Oberkochen, Germany). ImageJ software (National Institutes of Health, Bethesda, MD, USA) was used for the quantification of fluorescence signals for BDNF, DCX, and p-CaMK II.

### 4.10. Statistical Analysis

Data were expressed as the mean ± SEM. Significant differences of experiments with two groups were analyzed by the Student’s *t*-test. Experiments with three or more groups were subjected to a one-way ANOVA followed by the post-hoc Williams multiple comparison test.

## 5. Conclusions

Orally administered HMF promptly penetrated the BBB and ameliorated (1) CUMS-induced body weight loss, (2) CUMS-induced reductions in BDNF expression, (3) CUMS-induced reductions in neurogenesis, (4) CUMS-induced reductions in the level of phosphorylated CaMK II. The effects of HMF were inhibited by a MEK inhibitor. These results indicated that HMF acts as a novel antidepressant based on the “BDNF theory of depression”, and its effects appear to be dependent on ERK activation.

## Figures and Tables

**Table 1:** Chronic unpredictable stress procedure.

Day	Stress	Duration
1	Forced swim	15 min
2	Damp sawdust	24 h
3	Food deprivation	24 h
4	Restraint stress	2 h
5	Cage tilt	24 h
6	Electric footshock	0.5 mA, 10 s duration, 30 s interval, 20 shocks
7	Damp sawdust	24 h
8	Restraint stress	2 h
9	Food deprivation	24 h
10	Cage tilt	24 h
11	Electric footshock	0.5 mA, 10 s duration, 30 s interval, 20 shocks
12	Food deprivation	24 h
13	Restraint stress	2 h
14	Cage tilt	24 h

## References

[1] Barde Y.A. (1989). Trophic factors and neuronal survival. Neuron.

[2] Angelucci F., Brenè S., Mathé A.A. (2005). BDNF in schizophrenia, depression and corresponding animal models. Mol. Psychiatry.

[3] Björkholm C., Monteggia L.M. (2016). BDNF: A key transducer of antidepressant effects. Neuropharmacology.

[4] Knorr U., Vinberg M., Kessing L.V., Wetterslev J. (2010). Salivary cortisol in depressed patients versus control persons: A systematic review and meta-analysis. Psychoneuroendocrinology.

[5] Stetler C., Miller G.E. (2011). Depression and hypothalamic-pituitary-adrenal activation: A quantitative summary of four decades of research. Psychosom. Med..

[6] Duman R.S. (2005). Neurotrophic factors and regulation of mood: Role of exercise, diet and metabolism. Neurobiol. Aging.

[7] Duman R.S., Monteggia L.M. (2006). A neurotrophic model for stress-related mood disorders. Biol. Psychiatry.

[8] Cunha A.B., Frey B.N., Andreazza A.C., Goi J.D., Rosa A.R., Gonçalves C.A., Santin A., Kapczinski F. (2006). Serum brain-derived neurotrophic factor is decreased in bipolar disorder during depressive and manic episodes. Neurosci. Lett..

[9] Mao Q.Q., Huang Z., Zhong X.M., Xian Y.F., Ip S.P. (2014). Piperine reverses the effects of corticosterone on behavior and hippocampal BDNF expression in mice. Neurochem. Int..

[10] Demuyser T., Bentea E., Deneyer L., Albertini G., Massie A., Smolders I. (2016). Disruption of the HPA-axis through corticosterone-release pellets induces robust depressive-like behavior and reduced BDNF levels in mice. Neurosci. Lett..

[11] Aydemir C., Yalcin E.S., Aksaray S., Kisa C., Yildirim S.G., Uzbay T., Goka E. (2006). Brain-derived neurotrophic factor (BDNF) changes in the serum of depressed women. Prog. Neuro-Psychopharmacol. Biol. Psychiatry.

[12] Dwivedi Y., Rao J.S., Rizavi H.S., Kotowski J., Conley R.R., Roberts R.C., Tamminga C.A., Pandey G.N. (2003). Abnormal expression and functional characteristics of cyclic adenosine monophosphate response element binding protein in postmortem brain of suicide subjects. Arch. Gen. Psychiatry.

[13] Martinowich K., Manji H., Lu B. (2007). New insights into BDNF function in depression and anxiety. Nat. Neurosci..

[14] Gourley S.L., Wu F.J., Taylor J.R. (2008). Corticosterone regulates pERK1/2 Map kinase in a chronic depression model. Ann. N. Y. Acad. Sci..

[15] Shibata S., Iinuma M., Soumiya H., Fukumitsu H., Furukawa Y., Furukawa S. (2015). A novel 2-decenoic acid thioester ameliorates corticosterone-induced depression- and anxiety-like behaviors and normalizes reduced hippocampal signal transduction in treated mice. Pharmacol. Res. Perspect..

[16] Sawamoto A., Okuyama S., Yamamoto K., Amakura Y., Yoshimura M., Nakajima M., Furukawa Y. (2016). 3,5,6,7,8,3′,4′-Heptamethoxyflavone, a citrus flavonoid, ameliorates corticosterone-induced depression-like behavior and restores brain-derived neurotrophic factor expression, neurogenesis, and neuroplasticity in the hippocampus. Molecules.

[17] Okuyama S., Miyazaki K., Yamada R., Amakura Y., Yoshimura M., Sawamoto A., Nakajima M., Furukawa Y. (2017). Permeation of polymethoxyflavones into the mouse brain and their effect on MK-801-induced locomotive hyperactivity. Int. J. Mol. Sci..

[18] Willner P., Towell A., Sampson D., Sophokleous S., Muscat R. (1987). Reduction of sucrose preference by chronic unpredictable mild stress, and its restoration by a tricyclic antidepressant. Psychopharmacology.

[19] Monleon S., Parra A., Simon V.M., Brain P.F., D’Aquila P., Willner P. (1995). Attenuation of sucrose consumption in mice by chronic mild stress and its restoration by imipramine. Psychopharmacology.

[20] Willner P. (2017). The chronic mild stress (CMS) model of depression: History, evaluation and usage. Neurobiol. Stress.

[21] Grønli J., Bramham C., Murison R., Kanhema T., Fiske E., Bjorvatn B., Ursin R., Portas C.M. (2006). Chronic mild stress inhibits BDNF protein expression and CREB activation in the dentate gyrus but not in the hippocampus proper. Pharmacol. Biochem. Behav..

[22] Tianzhu Z., Shihai Y., Juan D. (2014). Antidepressant-like effects of cordycepin in a mice model of chronic unpredictable mild stress. Evid. Based Complement. Altern. Med..

[23] Furukawa Y., Okuyama S., Amakura Y., Watanabe S., Fukata T., Nakajima M., Yoshimura M., Yoshida T. (2012). Isolation and characterization of activators of ERK/MAPK from citrus plants. Int. J. Mol. Sci..

[24] Zhou D., Jin H., Lin H.B., Yang X.M., Cheng Y.F., Deng F.J., Xu J.P. (2010). Antidepressant effect of the extracts from Fructus Akebiae. Pharmacol. Biochem. Behav..

[25] Gu H.F., Nie Y.X., Tong Q.Z., Tang Y.L., Zeng Y., Jing K.Q., Zheng X., Liao D.F. (2014). Epigallocatechin-3-gallate attenuates impairment of learning and memory in chronic unpredictable mild stress-treated rats by restoring hippocampal autophagic flux. PLoS ONE.

[26] Porsolt R.D., Bertin A., Jalfre M. (1977). Behavioral despair in mice: A primary screening test for antidepressants. Arch. Int. Pharmacodyn. Ther..

[27] Petit-Demouliere B., Chenu F., Bourin M. (2004). Forced swimming test in mice: A review of antidepressant activity. Psychopharmacology.

[28] Lucassen P.J., Oomen C.A., Schouten M., Encinas J.M., Fitzsimons C.P., Juan J.C. (2016). Adult neurogenesis, chronic stress and depression. Adult Neurogenesis in the Hippocampus: Health, Psychopathology, and Brain Disease.

[29] Bathina S., Das U.N. (2015). Brain-derived neurotrophic factor and its clinical implications. Arch. Med. Sci..

[30] Davila D., Thibault K., Fiacco T.A., Agulhon C. (2013). Recent molecular approaches to understanding astrocyte function in vivo. Front. Cell. Neurosci..

[31] Quesseveur G., David D.J., Gaillard M.C., Pla P., Wu M.V., Nguyen H.T., Nicolas V., Auregan G., David I., Dranovsky A. (2013). BDNF overexpression in mouse hippocampal astrocytes promotes local neurogenesis and elicits anxiolytic-like activities. Transl. Psychiatry.

[32] Nibuya M., Morinobu S., Duman R.S. (1995). Regulation of BDNF and trkB mRNA in rat brain by chronic electroconvulsive seizure and antidepressant drug treatments. J. Neurosci..

[33] Paizanis E., Hamon M., Lanfumey L. (2007). Hippocampal neurogenesis, depressive disorders, and antidepressant therapy. Neural Plast..

[34] Vithlani M., Hines R.M., Zhong P., Terunuma M., Hines D.J., Revilla-Sanchez R., Jurd R., Haydon P., Rios M., Brandon N. (2013). The ability of BDNF to modify neurogenesis and depressive-like behaviors is dependent upon phosphorylation of tyrosine residues 365/367 in the GABA_A_-receptor γ2 subunit. J. Neurosci..

[35] Gerges N.Z., Aleisa A.M., Schwarz L.A., Alkadhi K.A. (2004). Reduced basal CAMKII levels in hippocampal CA1 region: Possible cause of stress induced impairment of LTP in chronically stressed rats. Hippocampus.

[36] Shi Y., Yuan Y., Xu Z., Pu M., Wang C., Zhang Y., Liu Z., Wang C., Li L., Zhang Z. (2012). Genetic variation in the calcium/calmodulin-dependent protein kinase (CaMK) pathway is associated with antidepressant response in females. J. Affect. Disord..

[37] Mazzucchelli C., Brambilla R. (2000). Ras-related and MAPK signalling in neuronal plasticity and memory formation. Cell. Mol. Life Sci..

[38] Wong J., Wislet-Gendebien S. (2013). Neurotrophin signaling and Alzheimer’s disease neurodegeneration-focus on BDNF/TrkB signaling. Trends in Cell Signaling Pathways in Neuronal Fate Decision.

[39] Bhatt D.K., Ramachandran R., Christensen S.L., Gupta S., Jansen-Olesen I., Olesen J. (2015). CGRP infusion in unanesthetized rats increases expression of c-Fos in the nucleus tractus solitarius and caudal ventrolateral medulla, but not in the trigeminal nucleus caudalis. Cephalalgia.

[40] Tao X., Finkbeiner S., Amold D.B., Shaywitz A.J., Greenberg M.E. (1998). Ca^2+^ influx regulates BDNF synthesis by a CREB family transcription factor-dependent mechanism. Neuron.

[41] Nagase H., Omae N., Omori A., Nakagawasai O., Tadano T., Yokosuka A., Sashida Y., Mimaki Y., Yamakuni T., Ohizumi Y. (2005). Nobiletin and its related flavonoids with CRE-dependent transcription-stimulating and neuritegenic activities. Biochem. Biophys. Res. Commun..

[42] Kawahata I., Yoshida M., Sun W., Nakajima A., Lai Y., Osaka N., Matsuzaki K., Yokosuka A., Mimaki Y., Naganuma A. (2013). Potent activity of nobiletin-rich Citrus reticulata peel extract to facilitate cAMP/PKA/ERK/CREB signaling associated with learning and memory in cultured hippocampal neurons: Identification of the substances responsible for the pharmacological action. J. Neural Transm..

[43] Luo L., Liu X.L., Li J., Mu R.H., Li Q., Yi L.T., Geng D. (2015). Macranthol promotes hippocampal neuronal proliferation in mice via BDNF-TrkB-PI3K/Akt signaling pathway. Eur. J. Pharmacol..

[44] Corrêa da Costa V.M., Mousovich-Neto F. (2016). Chronic Stress: Glucocorticoids and Metabolic Disturbances. J. Clin. Mol. Endocrinol..

[45] Zhou W., Yoshioka M., Yokogoshi H. (2009). Sub-chronic effects of *s*-limonene on brain neurotransmitter levels and behavior of rats. J. Nutr. Sci. Vitaminol..

[46] Okuyama S., Shimada N., Kaji M., Morita M., Miyoshi K., Minami S., Amakura Y., Yoshimura M., Yoshida T., Watanabe S. (2012). Heptamethoxyflavone, a citrus flavonoid, enhances brain-derived neurotrophic factor production and neurogenesis in the hippocampus following cerebral global ischemia in mice. Neurosci. Lett..

